# Structure-based Mechanistic Insights into Terminal Amide Synthase in Nosiheptide-Represented Thiopeptides Biosynthesis

**DOI:** 10.1038/srep12744

**Published:** 2015-08-05

**Authors:** Shanshan Liu, Heng Guo, Tianlong Zhang, Li Han, Pengfei Yao, Yan Zhang, Naiyan Rong, Yi Yu, Wenxian Lan, Chunxi Wang, Jianping Ding, Renxiao Wang, Wen Liu, Chunyang Cao

**Affiliations:** 1 State Key Laboratory of Bio-Organic and Natural Product Chemistry and Collaborative Innovation Center of Chemistry for Life Sciences, Shanghai Institute of Organic Chemistry, Chinese Academy of Sciences, 345 Lingling Road, Shanghai, 200032, China; 2 Institute of Biochemistry and Cell Biology, Shanghai Institutes for Biological Sciences, Chinese Academy of Sciences, 320 Yueyang Road, Shanghai, 200031, China

## Abstract

Nosiheptide is a parent compound of thiopeptide family that exhibit potent activities against various bacterial pathogens. Its C-terminal amide formation is catalyzed by NosA, which is an unusual strategy for maturating certain thiopeptides by processing their precursor peptides featuring a serine extension. We here report the crystal structure of truncated NosA_1-111_ variant, revealing three key elements, including basic lysine 49 (K49), acidic glutamic acid 101 (E101) and flexible C-terminal loop NosA_112-151_, are crucial to the catalytic terminal amide formation in nosiheptide biosynthesis. The side-chain of residue K49 and the C-terminal loop fasten the substrate through hydrogen bonds and hydrophobic interactions. The side-chain of residue E101 enhances nucleophilic attack of H_2_O to the methyl imine intermediate, leading to C_α_-N bond cleavage and nosiheptide maturation. The sequence alignment of NosA and its homologs NocA, PbtH, TpdK and BerI, and the enzymatic assay suggest that the mechanistic studies on NosA present an intriguing paradigm about how NosA family members function during thiopeptide biosynthesis.

Thiopeptides are a class of sulfur-rich, highly modified peptide antibiotics that are active against various drug-resistant bacterial pathogens. These antibiotics share a common ribosomally synthesized paradigm in biosynthesis, featuring conserved post-translational modifications of a precursor peptide to afford a family-characteristic framework in which a nitrogen-containing, six-membered ring is central to multiple azoles and dehydroamino acids. Many thiopeptides, including the bimacrocyclic members nosiheptide and thiostrepton (Fig. 1), possess a terminal amide moiety, formation of which, however, can proceed in completely different biosynthetic routes. In thiostrepton biosynthesis^1^,^2^, terminal amide formation involves an asparagine synthetase-like protein to incorporate an exogenous amino group arising from Gln, a precursor peptide-independent residue (where enzymes TsrB and TsrC catalyzes deesterification and amidation for thiostrepton maturation, respectively). In contrast, the amino group of the terminal amide moiety in nosiheptide is endogenous and derives from an extended Ser residue of the precursor peptide^3^,^4^. Dehydration of this residue at the early stage in the nosiheptide biosynthetic pathway generates enamide, and subsequent dealkylation requires the activity of NosA, which has recently been characterized as a new terminal amide synthase, leading to a C_α_-N bond cleavage for nosiheptide maturation with release of the co-product pyruvate. NosA catalyzed reaction is apparently distinct from those of known endogenously C-terminal amide-forming proteins, which typically catalyze an oxidative cleavage of C-terminal Gly-extended peptides accompanying glyoxylate production^5^,^6^,^7^,^8^. In this study, we provide the structural basis of NosA for investigation into its enzymatic mechanism.

## Results and Discussion

### Derivation of the truncated NosA_1-111_ variant and its X-ray crystal structure

The full-length NosA contains 151 amino acids in total. Within ten days, it degrades as a large fragment with a molecular weight about 12 KDa (supporting information, Fig. S1). This instability is likely due to the sequence (residues 106-151) at the C-terminus, which has a potential to form a flexible loop based on the secondary structure analysis (http://bioinf.cs.ucl.ac.uk/psipred/) (supporting information, Fig. S2). Thus, we truncated NosA by cutting off the residues at its C-terminus ten by ten, and constructed the pET28a plasmids containing the genes NosA_1-140_ (*i.e.*, residues 1-140), NosA_1-130_ (*i.e.*, residues 1-130), NosA_1-120_ (*i.e.*, residues 1-120), and NosA_1-111_ (*i.e.*, residues 1-111), and carried out their overexpression and purification as we did on wild-type (WT) NosA. By running SDS_PAGE gels (supporting information, Fig. S1), we found that, except NosA_1-111_, other NosA variants are still unstable (Among them, NosA_1-130_ is too unstable to be obtained in the process of purification).

We thus overexpressed NosA_1-111_ variant and its Se-Met form, and purified them to homogeneity for crystallization. The purified proteins were estimated to have a purity of >95%. The crystals grew in a cubic form. The diffraction of Se-Met NosA_1-111_ was extended to 2.40 Å resolution. Its crystals belong to the primitive cubic space group *P*4_1_32, with unit cell parameters of *a = b = c* = 143.3 Å. X-ray diffraction data sets of Se-Met NosA_1-111_ were processed using data in the resolution ranges 50.0–2.4 Å. The typical Matthews coefficient and solvent content were estimated as 3.94 Å^3^ Da^−1^ and 40.3%, respectively. The three-dimensional (3D) structure of NosA_1-111_ was determined using the single-wavelength anomalous-dispersion (SAD) method. The crystal parameters and data-collection statistics were summarized in Table 1.

Three monomers occupy one asymmetric unit (Fig. 2A,B), two of them form a dimer. One monomer constitutes a dimer with one monomer in an adjacent asymmetric unit. Each monomer is identical to the others with an RMSD value of 0.21 Å for the backbone C_α_ atoms in the secondary structural regions, consisting of four anti-parallel β-sheets (β1, β2, β3, and β4), three α-helices (α1, α2 and α3) and six loops (L1, L2, L3, L4, L5 and L6), which are arranged in the order of β1- L1- α1- L2- β2- L3- β3- L4- α2- L5- α3- L6- β4 (Fig. 2C). The anti-parallel β-sheets form a semicircular hydrophobic surface, the α-helices and loops are located on the outside of the circle. The first six or fewer residues at the N-terminus are invisible in all monomers, and the residues from 35 to 42 are also invisible in the two monomers that form a dimer conformation. This dimeric structure adopts a global fold, resembling an elliptic β-barrel of 28.9 Å in height, with diameters of 15 Å and 23 Å (Fig. 2A). The α-helices and loops surround the β-barrel.

**Figure 1:**
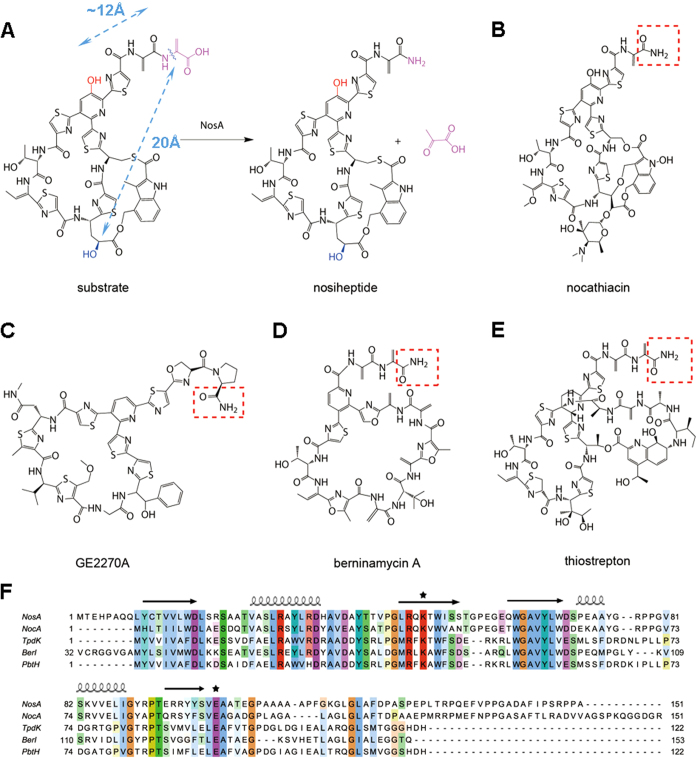
NosA and its homologs share a common mechanism on the post-modifications of thiopeptides. (**A**) The catalytic reaction for nosiheptide maturation by NosA. The –OH groups involved in the interactions with NosA are highlighted in red and blue, respectively. The C_α_-N bond cleavage site is marked in pink and a wavy line. The distances between the oxygen atoms of the –OH groups and the C_α_-N bond cleavage site in the substrate were measured as 12 Å and 20 Å, indicated by blue dotted lines, respectively. (**B**–**D**) the C-terminal amide formation of the thiopeptides similar to nosiheptide, such as nocathiacin (catalyzed by NocA), (**C**) GE2270A (catalyzed by TpdK or PbtH). (**D**) berninamycin A (catalyzed by BerI). (**E**) For comparison, thiostrepton maturation is done through deesterification and amidation by TsrB and TsrC, respectively. In (**B**–**E**), the products of the catalytic reactions were highlighted in red dashed boxes. (**F**) The sequence alignments of NosA and its homologs NocA, TpdK, PbtH and BerI. The conserved residues K49 and E101 were marked with stars on the top of the sequences. On the top of the amino acid sequence, the secondary structures of NosA were displayed, where arrows indicate β-sheets, and coils represent α-helices. The stars indicate the active sites observed in this report for enzymatic reaction.

**Figure 2:**
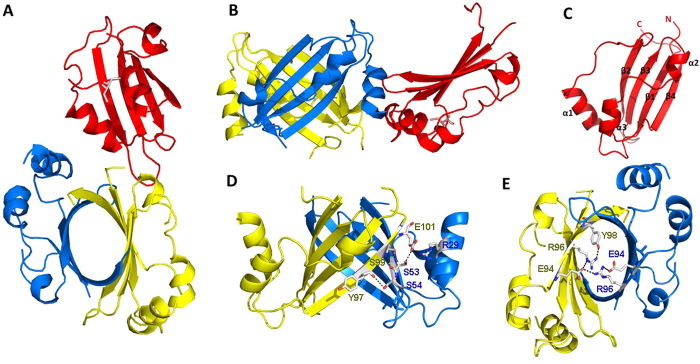
NosA_1-111_ overall fold. (**A**): vertical view, (**B**): lateral view) Ribbon representations of a NosA trimer observed in an asymmetric unit, monomers were highlighted in red, blue and yellow, respectively. (**C**) monomer conformation, N-terminal and C-terminal and secondary structures were marked; (**D**) residues forming the hydrogen bonds in the β4 strand and the β2’ strand outside of β-barrel; (**E**) the salt-bridge and hydrogen-bonds formation between R96 and E94’ within the β-barrel.

The β-barrel is formed through several hydrogen bonds between the residues of the β4 strand in one monomer and the β2’ strand in another monomer (Fig. 2D), including hydrogen bonds between the E101 carboxyl and S53’ –OH group or R29’ imide group, between the S99 backbone carboxyl and the S53’ backbone nitrogen, and between the S99 backbone nitrogen and the S53’ carboxyl; and a hydrogen bond between the Y97 backbone oxygen and the S54’ side-chain –OH group. The β-barrel is also stabilized by salt bridge within the β-barrel between the side chains of the R96 and E94’ residues (Fig. 2E). Among these residues, R29 and E101 are conserved in the NosA homologs (Fig. 1), indicating that they may be important for enzyme dimerization.

### The possible key sites for the catalytic reaction

**Figure 3:**
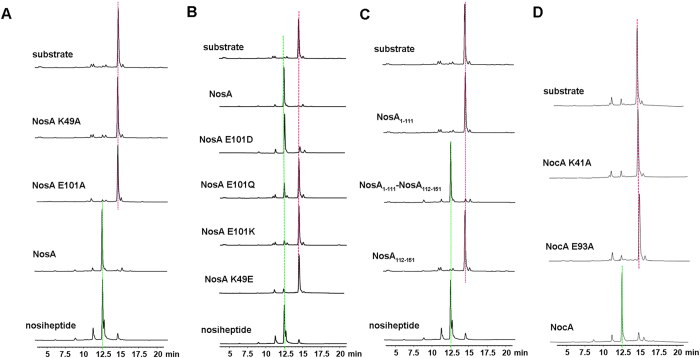
Enzymatic assay on the full-length NosA, NocA and their variants: (**A**) NosA and its K49A and E101A variants; (**B**) NosA and its E101D, E101Q, E101K and K49E variants; (**C**) NosA_1-111_, NosA_1-111_ mixed with NosA_112-151_ at mole ratio 1:1, and NosA_112-151_; (**D**) NocA and its K41A and E93A variants. In all cases, the substrate (top) and the product nosiheptide (bottom) were used as controls, highlighted in a dotted red line and green line, respectively.

To understand how NosA interacts with its substrate, we searched NosA structural homologs in protein data bank using DALI server^9^. The structures with Z-score higher than 7.0 were selected. They are heme-degrading enzymes^10^,^11^,^12^,^13^,^14^,^15^ or HapK protein involved in prodigiosin biosynthesis^16^, demonstrating that their ligands bind to the region outside of the β-barrel (supporting information, Fig. S3). Based on these observations, we assumed that the substrate of NosA might also bind to the region out of the β-barrel. To confirm this, we performed alanine-scanning mutagenesis assay on the conserved residues that are located at the entrances of the β-barrel (such as C11 and Q48), within the β-barrel (such as R29 and S53), outside of the β-barrel (such as Q48, K49, W51 and E101), or in the C-terminal loop (such as F122, D123, P124, S126, D128, P129, R132, P133, E135, F136, P138 and P139) in the full-length NosA, respectively. Then, we measured the catalytic activities of these variants by running enzymatic assay on HPLC system (Fig. 3A and supporting information, Fig. S4). Only K49A and E101A variants abolish the catalytic activities, indicating that K49 and E101 might be key elements for catalytic reaction. As shown in Fig. 3B, when E101 was replaced by D101, Q101 or K101, and residue K49 was mutated into E49, respectively, the E101K, K49E and E101Q variants abolish or significantly lose catalytic activities, while E101D retains the catalytic activity, indicating that the charged side-chains of E101 and K49 are critical to the catalytic reaction.

**Figure 4:**
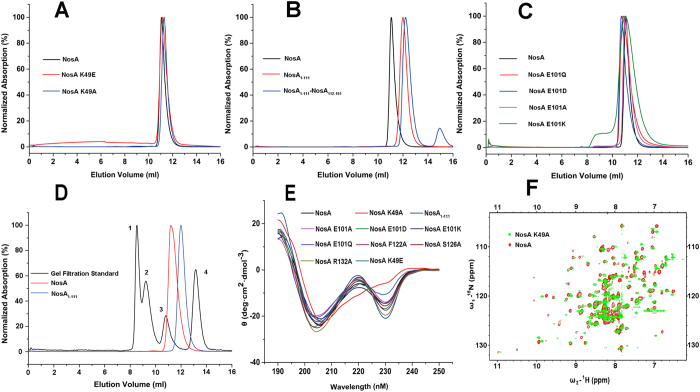
The folding and aggregation states of full-length NosA, NosA_1-111_ and its variants. (**A**–**D**) The aggregation states detected by size-exclusion chromatography assay; In (**D**), gel filtration protein standard markers were highlighted with arabic numerals 1, 2, 3 and 4, representing thyroglobulin with a molecular weight (MW) of 670KDa, γ-globulin with a MW of 158 KDa, Ovalbumin with a MW of 44 KDa, myoglobin with MW of 17 KDa and vitamin B12 with a MW of 1.35 KDa, respectively. (**E**) The folding of NosA and its variants detected by circular dichroism (CD) spectroscopies respectively; (**F**) The folding of NosA K49A variant further confirmed by two-dimensional NMR ^1^H-^15^N HSQC spectrum of the full-length NosA K49A variant (green), overlapped with that of wild-type NosA (red). In these two NMR experiments, the concentration of the WT NosA protein and its K49A variant is about 0.2 mM in NMR buffer.

Finally, we tested the possible effects on the folding and the aggregation state of NosA by these mutations by running the size-exclusion chromatography (SEC) assay, circular dichroism (CD) and nuclear magnetic resonance (NMR) spectroscopies. The results from the SEC assay suggested that the aggregation states of these variants were not affected by these mutations (Fig. 4A–C). Moreover, as shown in Fig. 4D, the retention time of the WT full-length NosA and the truncated NosA_1-111_ locates between gel filtration protein markers (3 and 4) with molecular weights of 44KDa and 17KDa, respectively, indicating that they are dimer in solution. Thus, the C-terminal loop is not helpful to NosA dimerization. However, the CD spectrum of the K49A variant looks much different from those of WT protein and other variants (Fig. 4E), and its 2D ^1^H-^15^N HSQC spectrum did not overlap well with that of WT NosA (Fig. 4F), indicating that the mutation from K49 to A49 might affect the folding of the protein. Thus, the loss of the catalytic activity of K49A might also result from the changes in the folding of the protein.

### NosA may function as a dimer

It was reported that the removal of two –OH groups on the substrate (highlighted in red and in blue in Fig. 1, respectively) (by knockouting the genes *nosC* and *nosB* in nosiheptide biosynthesis responsible for these –OH groups formation, respectively) made NosA completely lose the catalytic activities^17^, suggesting that these two –OH groups in the substrate are important for the catalytic reaction. Interestingly, as shown in supporting information, Fig. S5, in the current crystal structure of NosA_1-111_, the intra-molecule distance between the oxygen atom in the side-chain of E101 and the nitrogen atom in the side-chains of K49 is approximately 26 Å, much longer than the corresponding inter-molecular distance (13.6 Å) between these two atoms. The latter is close to that (~12 Å) between the –OH group in the pyridine ring (highlighted in red, Fig. 1A) and the C_α_-N bond cleavage site in the substrate (measured from the crystal structure of nosiheptide in complex with ribosomal subunit, pdb code 2ZJP, in which the distance between –OH group (highlighted in blue, Fig. 1A) and the C_α_-N bond cleavage site is 20 Å)^18^. Thus, we suggest that NosA may function as a dimer to catalyze the maturation reaction of nosiheptide, which is consistent with the dimer conformation of the full-length NosA and its truncated NosA_1-111_ variant detected by SEC assay above.

### NosA_112-151_ is crucial to the catalytic reaction

As we mentioned above, to get a stable form of NosA, we prepared several truncated NosA variants. Among them, NosA_1-111_ is the most stable. However, the enzymatic assay performed on the HPLC system indicates that NosA_1-111_ has no catalytic activity (Fig. 3C), revealing that the C-terminal loop NosA_112-151_ is extremely important to the catalytic reaction. To probe whether or not the catalytic efficiency of NosA_1-111_ can be rescued, we directly mixed NosA_1-111_ with NosA_112-151_ at mole ratio of 1:1, and performed the enzymatic assay again. The results demonstrate that the catalytic efficiency of the N-terminal NosA_1-111_ is partially recovered by the C-terminal NosA_112-151_. Further enzymatic parameter measurements suggest that the mixture of NosA_1-111_ and NosA_112-151_ has a catalytic efficiency of *k*_cat_/*K*_m_ = 4.93 × 10^−3^ min^−1^ μM^−1^ (where the catalytic power *k*_cat_ = 3.63 ± 0.7 min^−1^, and the Michaelis constant *K*_m_ = 736.1 ± 173.7 μM, respectively), decreased by approximately 2000-fold, compared to the full-length NosA (the catalytic efficiency *k*_cat_/*K*_m_ = 9.85 min^−1^ μM^−1^, where *k*_cat_ = 728.1 ± 144.7 min^−1^, and *K*_m_ = 73.9 ± 43.5 μM). This observation may be due to weak interaction between NosA_1-111_ and NosA_112-151_ (*K*_D_ = 2.4 ± 1.2 mM measured by ITC binding assay, supporting information, Fig. S6). In the case of NosA_1-111_ mixed with the peptide NosA_112-151_, the Michaelis constant *K*_m_ (the binding affinity of the substrate to the enzyme) is reduced by ten-fold, leading to a much weaker catalytic power (*k*_cat_ is decreased by 200-fold) than that of full-length NosA, suggesting that NosA_1-111_ might coordinate with NosA_112-151_ to interact with the substrate. Therefore, by mixing with NosA_112-151_, the catalytic activity of NosA_1-111_ is only slightly recovered with a significant drop in catalytic efficiency.

**Figure 5:**
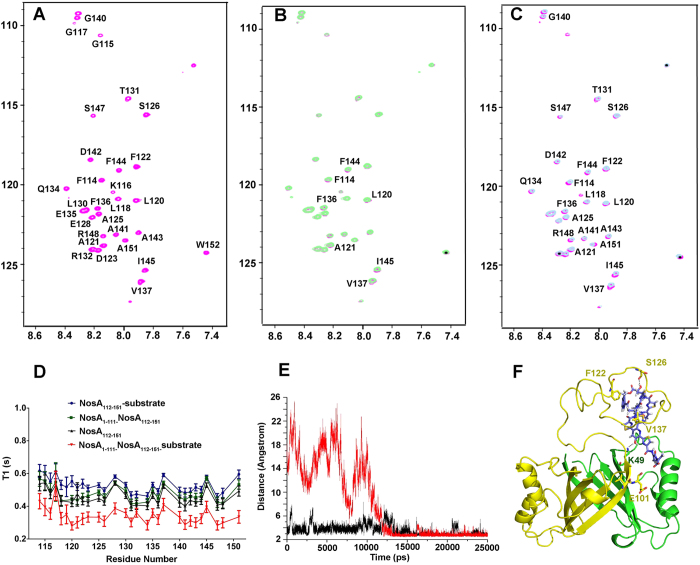
NosA_1-111_ coordinates with NosA_112-151_ to interact with the substrate. (**A**) ^1^H-^15^N HSQC spectra acquired on free NosA_112-151_, highlighted with NMR signal assignment of residues; (**B**) ^1^H-^15^N HSQC spectrum of NosA_112-151_ in complex with the substrate (in green) was overlapped with that of free NosA_112-151_ (in pink); the signals with chemical shifts changes were marked; (**C**) ^1^H-^15^N HSQC spectrum of NosA_112-151_ in complex with NosA_1-111_ and substrate (in grey), overlapped with that of free NosA_112-151_ (in pink); the signals with chemical shifts changes were marked; In all cases of (**A**–**C**), the concentration of NosA_112-151_ was about 0.2 mM in NMR buffer. (**D**) Relaxation time T_1_ measurements of backbone atoms of each residue in ^15^N-labeled NosA_112-151_ in its free state (black), in complex with NosA_1-111_ (green), in complex with the substrate (blue), in complex with NosA_1-111_ and substrate (red), respectively. (**E**) Two hydrogen bonds between NosA and the substrate are formed, supported by the distance changes during MD simulation trajectory. The change in the distance between nitrogen atom of the side-chain of K49 and oxygen atom in –OH group in pyridine of the substrate is shown in black, and the change in the distance between the backbone oxygen of S126 and oxygen atom in –OH group in the substrate highlighted in blue in Fig. 1 is shown in red, respectively. (**F**) The last snapshot of the MD simulation trajectory, where the two monomers of NosA were displayed in green and yellown ribbon modes, respectively. The substrate was displayed in cyan-sticks mode. The main residues contributing to the protein-substrate interactions, including K49 and E101 in NosA_1-111_ and residues in NosA_1-120_, were also shown in sticks mode. The two hydrogen-bonds between NosA and substrate were displayed in dotted lines.

To confirm this hypothesis, we performed the following biochemical assay. The CD spectrum of NosA_112-151_ reveals that the NosA_112-151_ peptide is disordered in its free state (supporting information, Fig. S7-A). Upon mixing with the N-terminal NosA_1-111_, the cross-peaks of the ^1^H-^15^N spectrum acquired on NosA_112-151_ are still not dispersed, mainly located in the region between 8.0 ppm and 8.5 ppm, similar to the observation in ^1^H-^15^N HSQC spectrum acquired on free NosA_112-151_ (Fig. 5A) (both spectra overlapped very well in supporting information, Fig. S7-B). This observation suggests that the C-terminal NosA_112-151_ peptide is still folded as a random coil conformer upon being mixed with NosA_1-111_. Moreover, the cross-peaks of the ^1^H-^15^N spectra acquired on NosA_112-151_ did not shift (supporting information, Fig. S7-B), indicating that NosA_1-111_ does interact very weakly with NosA_112-151_, consistent with the measurement of the binding affinity of NosA_1-111_ to NosA_112-151_ by ITC assay (supporting information, Fig. S6). Moreover, Titrating the substrate into ^15^N-labeled NosA_112-151_ solution only results in slight shift of several cross-peaks in ^1^H-^15^N HSQC of NosA_112-151_, suggesting that individual NosA_112-151_ interacts with the substrate weakly (Fig. 5B). Adding the substrate into the mixture of NosA_1-111_ and NosA_112-151_ leads to obvious, but still small chemical shift changes in the ^1^H-^15^N spectra (Fig. 5C), indicating that NosA_1-111_ may coordinate with NosA_112-151_ to interact with the substrate, consistent with the measurements of the *k*_cat_ and *K*_m_ values above. To further confirm this conclusion, we measured the dynamic properties of the backbone atoms (relaxation time T_1_ and T_2_ and ^15^N-^1^H NOE values) of free NosA_112-151_, and of NoxA_112-151_ mixed with NosA_1-111,_ and of NoxA_112-151_ mixed with the substrate, and of NoxA_112-151_ mixed with both NosA_1-111_ and the substrate, respectively. The T_1_ values of the backbone atoms of NosA_112-151_ mixed with the substrate and the N-terminal NosA_1-111_ are the smallest among these cases (Fig. 5D), indicating that the conformation of the NosA_112-151_ peptide in the ternary complex is the most rigid among these cases. This observation reveals that the flexible conformation of NosA_112-151_ may be fixed in the presence of NosA_1-111_ and the substrate.

**Figure 6:**
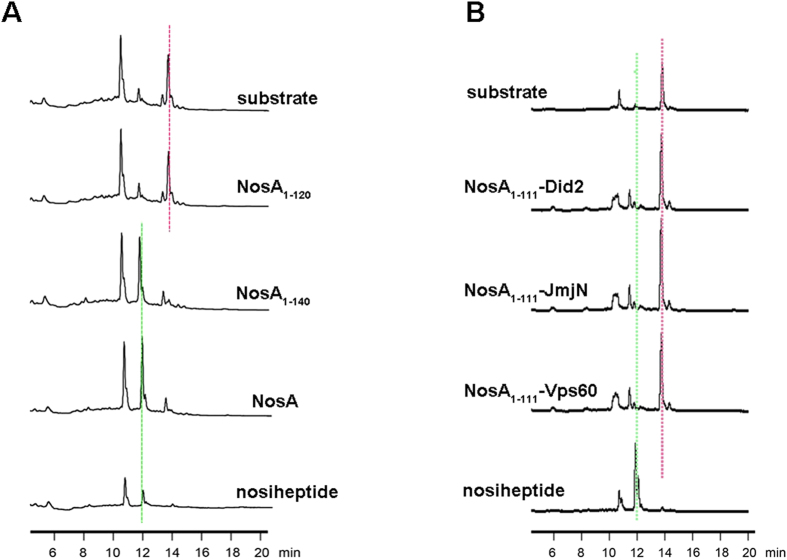
The enzymatic assay performed on HPLC systems: (**A**) from top to bottom, only substrate as a control, NosA_1-120_, NosA_1-140_ variants, full-length NosA, and the catalytic reaction product nosiheptide used as another control; (**B**) from top to bottom, only substrate as a control, NosA_1-111_ plus Did2, NosA_1-111_ plus JmjN from KDM5C, NosA_1-111_ plus Vps60, and the catalytic reaction product nosiheptide used as another control. In (**A**,**B**), the controls substrate and the product nosiheptide were indicated by pink and green dotted line, respectively.

To probe whether the whole sequence of NosA_112-151_ affects the catalytic reaction, we measured the catalytic activities of the truncated NosA variants with different length, and found that the NosA_1-140_ variant maintained the catalytic activity almost similar to the full-length NosA, whereas NosA_1-120_ significantly lost the catalytic activity (Fig. 6A), revealing that the residues from 120 to 140 (*i.e*., A_121_FDPASPEPLTRPQEFVPPG_140_) of NosA_112-151_ are important to the catalytic reaction. To investigate whether the sequence and the coiled-coil conformation of NosA_112-151_ are specific to the catalytic reaction, we replaced NosA_112-151_ in the mixture by three randomly-selected peptides (Did2, Vps60 and JmjN) available in the lab^19^,^20^,^21^,^22^, respectively, and tested the catalytic activities of these mixtures, all displaying no catalytic activities at all (Fig. 6B). These observations suggest that the sequence and the flexible loop conformation of the NosA_112-151_ variant is crucial to the catalytic reaction.

### NosA_1-111_ coordinates with NosA_112-151_ to bind the substrate

Since the substrate is not able to dissolve in solution, it’s impossible for us to get the crystals of the enzyme NosA in complex with the substrate. We also failed to get the crystals of NosA_1-111_ in complex with NosA_112-151_. Thus, to understand how the substrate interacts with NosA, ligand docking, homology modeling and molecular dynamics (MD) simulation were conducted, respectively. During MD simulation trajectory, we observed that: (1) the C-terminal NosA_112-151_ loop wrapped the substrate after 20–25 ns (supporting information, Fig. S8); (2) The hydrogen bond between the backbone carbonyl oxygen atom of residue S126 and the oxygen atom in the –OH group next to the carbonyl group on the substrate (highlighted in blue in Fig. 1A) was formed around 15 ns, and was maintained during the next 10 ns of simulation (Fig. 5E). We assumed that the formation of this hydrogen bond might induce the C-terminal loop to bend and wrap the substrate. The second hydrogen bond was formed between K49 side-chain in NosA and the -OH group in pyridine ring of the substrate, because the distance between them was kept less than 4 Å (Fig. 5E). These observations are supported by the fact that the catalytic power of NosA was completely abolished after removing these two hydroxyl groups in the substrate^17^. Moreover, the –OH group in pyridine ring is obviously more acidic than –OH group next to the carbonyl group in the substrate, which could interpret why the side-chain of residue K49 of NosA easily interacts with the –OH group in pyridine ring. (3) Residues involved in the protein-substrate interaction mainly locate at residues 120–140 on the C-terminal loop (Fig. 5F), consistent with the results from the enzymatic assay above. (4) The distance between the side-chain carboxylic oxygen atoms of E101 and the carbon atom at the C_α_-N bond cleavage site on the substrate is kept larger than 5 Å, smaller than 12 Å during the MD simulation (data not shown).

### The proposed mechanism for NosA catalytic amidation reaction

**Figure 7:**
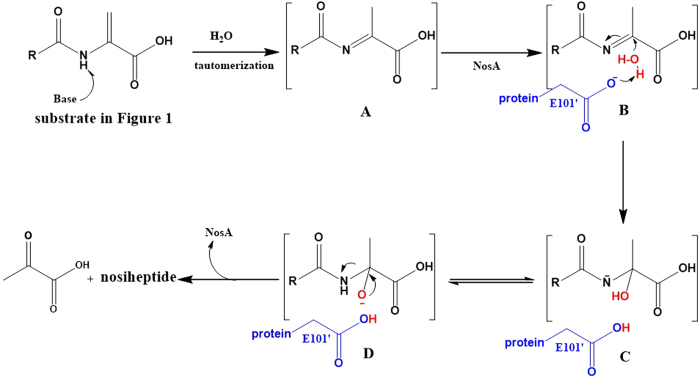
The proposed mechanism of NosA to catalyze nosiheptide maturation.

Taken all results above together, we proposed the following catalytic mechanism (Fig. 7): (1) The terminal dehydroalanine unit is tautomerized to methyl imine intermediate A in basic buffer condition, supported by the previous studies on thiostrepton synthesis^23^,^24^, where similar reaction is initiated by Et_2_NH; (2) The substrate is fixed into the active sites by hydrogen-bond and hydrophobic interactions between the substrate and residue K49, the C-terminus of NosA, generating intermediate B, supported by the structural and MD studies above; (3) The negatively charged side-chain of E101’ interacts with one molecule H_2_O, supported by the findings that several water molecules exist close to E101’ in the crystal structure; (4) The nucleophilic attack by H_2_O to methyl imine produces intermediates C and D, leading to the final Cα-N bond cleavage to yield nosiheptide and pyruvate.

### NosA family members catalyze the amidation reaction through a similar way

Most importantly, it was reported that NosA homologs could also catalyze the terminal amide formation of some thiopeptides (Fig. 1). For example, NocA (64% identity to NosA) catalyzes the formation of nocathiacin^25^, TpdK (34% identity to NosA) and PbtH (44% identity to NosA) catalyzes the final formation step in GE2270A biosynthesis^26^,^27^, BerI (48% identity to NosA) catalyzes the final step of berninamycin A in its biosynthesis^28^. Among these enzymes, the residues K49 and E101 are conserved, corresponding to residues K41 and E93 in NocA. Thus, we made the NocA K41A and E93A variants, and measured their catalytic activities on the same substrate (Fig. 3D). The results indicate that either the NocA K41A or E93A variant loses catalytic activities, revealing that NosA and its family members might share a common mechanism to catalyze the terminal amidation reaction.

In conclusion, we characterized three key elements (basic lysine, acidic glutamic acid and flexible C-terminal loop) for the terminal amide formation in nosiheptide-represented thiopeptide biosynthesis, these mechanistic studies on how NosA works present an intriguing paradigm about how NosA family members function during thiopeptide biosynthesis.

## Methods

### The expression and purification of NosA, NocA and their variants

The ORFs of full-length NosA (151 amino acids in total), truncated NosA variants, including the truncated NosA_1-111_ (*i.e*., residues 1-111), NosA_1-120_ (*i.e*., residues 1-120), NosA_1-130_ (*i.e*., residues 1-130), NosA_1-140_ (*i.e*., residues 1-140), and NocA (151 amino acids in total) were engineered into a pET28a vector with a His_6_-tag using *Nhe*I and *Hin*dIII restriction sites. Site-directed mutagenesis was performed using a QuikChange site-directed mutagenesis kit (Stratagene Inc.). The ORF of the C-terminal NosA_112-151_ (*i.e*., residues 112-151) was engineered into a pSMT3 vector with a SUMO tag, which can be removed using ULP1 protease. The constructs were verified by DNA sequencing, and the plasmids were transformed into *Escherichia coli* BL21(DE3) competent cells. The transformed cells were grown at 310 K in a Luria Broth (LB) medium containing 50 μg ml^−1^ kanamycin and were induced (24 h, 291 K) by the addition of 0.1 mM isopropyl-d-thio-b-D-galactopyranoside (IPTG) when the OD_600_ value was measured as 0.5 - 0.6. The cells were harvested and resuspended in lysis buffer (50 mM phosphate, pH 7.5, 500 mM NaCl), lysed with 10 μg ml^−1^ PMSF by sonication on ice. The lysates were clarified by centrifugation (30 min, 16,000 rpm), and the soluble proteins were purified by nickel-affinity chromatography (GE Healthcare) though a linear gradient of 0–500 mM imidazole in the lysis buffer. Protein fractions were collected and dialyzed twice at 277 K against the lysis buffer. To remove the His_6_-tag, the fusion proteins were digested with thrombin protease (ULP1 protease for NosA_112-151_) overnight at 4 °C, followed by running a second nickel column.

The fractions were collected and further purified by running gel-filtration chromatography on a Superdex 75 column (GE Healthcare) with a buffer containing 25 mM phosphate, pH 7.5, and 50 mM NaCl. Finally, the peak fractions containing proteins were concentrated to 50 mg ml^−1^ using an ultra-centrifugal filter tube (Millipore) and were used for crystallization (50 mM Tris-HCl, pH 7.5 50 mM NaCl for crystallization), NMR experiments, stability testing experiment by running SDS-PAGE gels, or kinetic experiments. The pure protein fractions were further verified by running an SDS-PAGE gel and electrospray mass spectrometry. The protein concentrations were estimated from the absorbance at 280 nm with their corresponding absorption coefficients.

The truncated NosA_1-111_ variant has one Met residue at its N-terminal sequence. Thus, for crystallization, the selenomethionine NosA_1-111_ (Se-Met NosA_1-111_) was successfully expressed in M9 medium using the reported methionine-pathway inhibition protocol^29^,^30^ and was purified as performed on native NosA_1-111_ above.

For NMR experiments, the ^15^N-labeled NosA_112-151_, the full-length NosA and its K49A variant, and the ^13^C- and ^15^N-labeled NosA_112-151_ were expressed in M9 medium containing ^15^NH_4_Cl and/or ^13^C-glucose as the nitrogen and carbon source, respectively.

### NosA_1-111_ crystallization and its X-ray data collection

Initial crystallization trials were performed at 293 K with Crystal Screen HT and Index HT kits in 96-well plates using the sitting-drop vapor-diffusion method (Hampton Research). For refinement of the crystallization conditions, 1 μl of protein solution was mixed with an equal volume of reservoir solution and equilibrated against 0.5 ml of the reservoir solution at 293 K in 24-well plates using the sitting-drop vapor-diffusion method. Crystals of Se-Met NosA suitable for X-ray analysis were obtained under the following conditions: 0.02 M magnesium chloride hexahydrate, 0.1 M HEPES pH 6.0 - 9.0, and 22% w/v polyacrylic acid 5100.

The crystals were picked up in a nylon loop (Hampton Research) and mounted in liquid nitrogen for flash-cooling. X-ray diffraction data were collected at beamline BL17U of the Shanghai Synchrotron Radiation Facility (SSRF, China) using a MAR CCD MX-225 detector. The wavelength of the radiation was 0.9794 Å, and the distance between the crystal and the detector was 400 mm. The exposure time for each frame was 1 s with a 1^o^ oscillation, and 360 frames were collected. The data were indexed, integrated and scaled using the HKL-2000 program suite^31^. The structure was solved using the single-wavelength anomalous dispersion method implemented in Phenix^32^, which identified 3 distinct Se atoms and yielded a figure of merit of 0.22. Model building was performed using Coot^33^. Structure refinement was carried out using Phenix and Refmac5^32^,^34^. Structure analysis was carried out using programs in CCP4^35^. The figures were generated using Pymol (http://www.pymol.org). The statistics of the structure refinement and the quality of the final structure models are summarized in Table 1.

### The enzymatic assay on HPLC system

To assay the activities of the full-length NosA, NocA and their variants, the truncated N-terminal NosA_1-111_ variant, the mixture of NosA_1-111_ with the C-terminal NosA_112-151_ peptide, each protein solution (including the full-length NosA, NocA or their mutants, or the mixture of NosA_1-111_ with NosA_112-151_ at mole ratio of 1:1.) was diluted to 1 mg/ml in buffer A (50 mM Glycine buffer, pH 9.0). To avoid degrading, all samples are used after purification was finished. The substrate was first dissolved in DMSO, and then diluted to 1 mg/ml by buffer A. For simple comparison, 5 μL of diluted protein solution and 5 μL of substrate solution were added into 40 μL of buffer A, respectively, and then was incubated at 303 K for 60 min. To quench the reaction, 5 μL of methanol were added. To determine whether the product nosiheptide was generated or not, HPLC analysis was performed by running C-18 reverse phase column (Agilent 1100) with a MeCN/H_2_O gradient mobile phase. The wavelength of nosiheptide detection was specified as 254 nm.

To investigate the importance of the sequence and the coiled-coil conformation of the C-terminal NosA_112-151_ in the catalytic reaction, three randomly selected peptides (Did2, Vps60 and JmjN from KDM5B) available in the lab were used to replace NosA_112-151_, and to mix with the N-terminal NosA_1-111_ variant. Both Did2 (176-204 aa, with an amino acid sequence as follows: NVPEIKAKEV NVDDEKEDKL AQRLRALRG) and Vps60 (128-186 aa, with an amino acid sequence as follows: INIDKLQDMQ DEMLDLIEQG DELQEVLAMN NNSGELDDIS DAELDAELDA LAQEDFTLP) peptides were reported in ESCRT-III system involved in multivesicular bodies (MVB) pathway^21^,^22^, with a helix conformation and a random-coiled conformation in their free states, respectively. The JmjN peptide (with an amino acid sequence as follows: ECPVFEPSWA EFRDPLGYIA KIRPIAEKSG ICKIRPPAD) locates at the N-terminal histone H3K4me3/2 demethylase KDM5B^19^,^20^, with a regular conformation. NosA_1-111_ was first mixed with each peptide at mole ratio 1:1, then the assay was performed as we did on the mixture of NosA_1-111_ and NosA_112-151_.

For *k*_cat_ and *K*_m_ measurements of the full-length NosA, a time course was carried out to determine the initial rate in 50 mM Glycine-NaOH buffer (20 μL, pH = 9.0) that contained 10 nM NosA and 200 μM substrate. The reactions were initiated by the addition of NosA into diluted substrate solution, incubated at 303 K, and then terminated by adding 50 μL of methanol into the solution at 2, 5, 10, 15, 30, 60, 120, and 240 min, respectively. The samples were subjected to the same workups and HPLC analysis as described above. The production of product (*i.e*., the intensity of the peak was used to quantify the nosiheptide), linear with respect to time during 0–5 min, was fitted into a linear equation to obtain the initial velocity. To determine the kinetic parameters for the conversion of substrate to product, the reactions were carried out at 303 K for 5 min in each 20 μl of the mixture that contained 10 nM NosA, 50 mM Glycine-NaOH (20 μL, pH = 9.0), and the substrate concentration varying at 50, 100, 150, 200, 250, and 500 μM, respectively.

For *k*_cat_ and *K*_m_ measurement of NosA_1-111_ mixing with NosA_112-151_, a time course was carried out to determine the initial rate in 50 mM Glycine-NaOH (20 μL, pH = 9.0) buffer that contained 4 μM NosA_1-111_, 4 μM of NosA_112-151_ and 200 μM substrate. The reactions were initiated by the addition of mixture of NosA_1-111_ and NosA_112-151_ into the diluted substrate solution, incubated at 303 K, and then terminated by adding 50 μL of methanol into the reaction solution at 2, 5, 10, 15, 30 min, respectively. The samples were subjected to similar HPLC analysis as described above. The production of product, linear with respect to time during 0–2 min, was fitted into a linear equation to obtain the initial velocity. To determine the kinetic parameters for the conversion of substrate to product, the reactions were carried out at 303 K for 5 min in each 20 μl of the mixture that contained 4 μM NosA_1-111_, 4 μM NosA_112-151_, 50 mM Glycine-NaOH (20 μL, pH = 9.0), and the substrate concentration varying at 50, 100, 150, 200, 250, and 500 μM, respectively.

All assays were performed in duplicate. Each conversion was analyzed by HPLC as described above. The resulting initial velocities were then fitted to the Michaelis-Menten equation using GraphPad Prism 5 to extract *K*_m_ and *k*_cat_ parameters.

### Molecular modeling of complexation between full-length NosA with its substrate

To understand how the substrate interacts with NosA, as the first step, the substrate molecule was docked onto the crystal structure of NosA_1-111_. A distance-restrained docking was performed based on two observations. Firstly, the results of enzymatic assay indicate that the C-terminal NosA_112-151_ loop is important for catalytic reaction, which may be due to the stabilizing effect of the C-terminus on the complex structure. Due to the absence of C-terminus, it is probably difficult to generate a rational binding mode for the substrate and NosA_1-111_ by an unrestrained docking. Secondly, based on the information that the residues K49 and E101 play a vital role in maintaining the catalytic activity of NosA, the distances between the side-chain amino group of K49 and the oxygen atom of the –OH group in the pyridine ring of the substrate and between the side-chain carboxylic oxygen atoms of E101 and the nitrogen atom at the C_α_-N bond cleavage site on the substrate were restricted within 2.5–3.5 Å during molecular docking. All ligand docking jobs were performed using the GOLD software suite^36^.

As the second step, a structural model of C-terminal NosA_112-151_ was derived through homology modeling. Our homology search found that the C-terminus of the HTLV-II matrix protein (PDB code 1JVR)^37^, which is also an irregular loop, had a sequence identity of 37.5% to the C-terminal NosA_112-151_ peptide. Thus, it was used as the template to build a model of the NosA C-terminus. The homology modeling job was completed by using the MOE software (version 2013) (Chemical Computing Group Inc., Montreal, Quebec, Canada). The resulting structure was adjusted manually to avoid steric hindrance between C-terminus and the rest part of the complex. Simultaneously, the main chain of C-terminus should stay as close as possible to the –OH group next to the carbonyl group of the substrate (Fig. 1A, highlighted in blue) according to the fact that the catalytic activity was abolished due to the deletion of this –OH group^17^. Finally, the adjusted structure was assembled onto the crystal structure of the N-terminal NosA_1-111_.

The third step was to refine the binding mode of the substrate to NosA, which was generated by molecular docking as described above, through molecular dynamics (MD) simulation. Our MD simulations were performed by using the AMBER software (version 12)^38^. The docking model was supplied as the initial configuration for MD simulation. The AMBER FF12SB force field^39^ was applied. The partial charges were calculated with the Gaussian 09 software^40^ at the HF/6-31G(d) level and were further processed by the RESP model^41^. The complex structure was soaked in a TIP3P^42^ water box with a margin of 10 Å at each direction. The whole system was neutralized by addition of a proper number of counterions (Na^+^). The system was gradually heated to 300 K over 100 ps. Then, a total of 25 ns simulation was performed at a 2-fs interval under a constant temperature of 300 K and a constant pressure of 1 atm. During simulation, all covalent bonds containing hydrogen atoms were constrained with the SHAKE algorithm^43^. Long-range electrostatic effects were modeled using the particle-mesh-Ewald method^44^ with a cutoff of 12 Å. The Langevin equilibration scheme^45^ was used to control and equalize the temperature. Snapshots were saved every 500 fs during simulation. The resulting MD trajectories were analyzed using the PTRAJ module and visualized with the VMD software^46^.

### Circular dichroism spectra of NosA_112-151_, full-length NosA and its variants

To probe the folding of full-length NosA and its variants, and the C-terminal NosA_112-151_ peptide, circular dichroism (CD) experiments were performed at 298 K on a JASCO-715 spectropolarimeter (Jasco International Co., Tokyo, Japan). Data were collected at 0.1-nm intervals at a scan speed of 20 nm/min, a 1-nm bandwidth, and a 0.25-s response time from 250 to 190 nm. Circular quartz cells of 1- and 0.1-cm path lengths were used for the far-UV regions. The CD intensities are expressed as the molar residue ellipticities given in units of degrees cm^2^ mol^−1^. The concentration of protein or peptide was about 20 μm. The buffer conditions used for running the CD spectra were 50 mM sodium phosphate (pH 7.0).

### NMR spectroscopy and analysis

To assign NMR resonances of the backbone atoms of C-terminal NosA_112-151_, the NMR sample was made containing 1.5 mM uniformly ^13^C/^15^N-labelled NosA_112-151_ in NMR buffer (50 mM Na_2_HPO_4_, 50 mM NaCl, 0.01% NaN_3_, pH 7.0 and 10% D_2_O). All NMR experiments for assigning the ^1^H, ^13^C and ^15^N backbone atoms (including 2D ^15^N-^1^H HSQC, 3D HNCA, HNCO, HN(CO)CA, HNCACB, CBCA(CO)NH) were performed at 298 K on a Varian Unity Inova 600 NMR spectrometer equipped with a triple resonances cryoprobe and pulsed field gradients.

To probe the effects on the folding of NosA by the mutation from K49 to A49, we first run circular dichroism (CD) spectra on both wild-type (WT) NosA and its K49A variant. The results from CD spectra indicate that the folding of K49A looks much different from that of WT full-length protein. To double check these changes on the folding of the K49A variant, ^1^H-^15^N HSQC spectra were acquired on WT full-length NosA and its K49A variant at 293 K in NMR buffer (50 mM Na_2_HPO_4_, 50 mM NaCl, 0.01% NaN_3_, pH 7.0 and 10% D_2_O).

To study interactions among NosA_1-111_, NosA_112-151_ and the substrate, ^1^H-^15^N HSQC spectra were acquired at 293 K on 1 mM ^15^N-labeled NosA_112-151_ in its free state, in complex with unlabeled NosA_1-111_ (the mole ratio of them is NosA_1-111_ : NosA_112-151_ = 1: 1.2), or in complex with unlabeled NosA_1-111_ and the substrate (the mole ratio of them is NosA_1-111_ : NosA_112-151_ : substrate = 1: 1.2 : 1.2) in NMR buffer (50 mM Na_2_HPO_4_, 50 mM NaCl, 0.01% NaN_3_, pH 7.0 and 10% D_2_O).

To determine the flexibility of the NosA_112-151_ in the presence of NosA_1-111_ and the substrate, the measurements of the relaxation times, T_1_ and T_2_ and ^15^N-^1^H NOEs of the backbone atoms of the NosA_112-151_ were performed at 293 K in NMR buffer (50 mM phosphate, 50 mM NaCl, 0.01% NaN_3_, pH 7.0 and 10% D_2_O) at 293 K for NosA_112-151_ on a Varian INOVA 600-MHz spectrometer. Ten different values for the relaxation delay were used for the T_1_ (delays 60, 80, 100, 140, 240, 360, 540 and 760 ms) and T_2_ (Carr-Purcell-Meiboom-Gill mixing times of 10, 30, 50, 70, 90, 110, 150, 170 and 190 ms) relaxation experiments. The T_1_ and T_2_ values were obtained by nonlinear squares fits using the program Prism 5. The ^15^N-^1^H NOE values were calculated as the ratio of the intensities of the paired ^15^N-^1^H correlation peaks from interleaved spectra acquired with and without ^1^H pre-saturation during a recycle time of 3 s.

All NMR spectra were processed with the NMRPipe program^47^ and analyzed using Sparky 3 (http://www.cgl.ucsf.edu/home/sparky/).

### Size-exclusion chromatography (SEC) assay

To probe effects on the aggregation state of NosA by the mutations, the size-exclusion chromatography assay was performed on a Superdex 75 column (10/300 GL) (GE Healthcare), which was previously equilibrated with buffer B (50 mM Tris-HCl, pH 7.5, 50 mM NaCl).

### Isothermal titration calorimetry (ITC) binding assay

To investigate the binding affinity of the N-terminal NosA_1-111_ with the C-terminal NosA_112-151_, the isothermal titration calorimetry (ITC) binding assay was performed. An ITC-200 microcalorimeter (GE Healthcare) was used with a buffer containing 20 mM Tris-HCl, 150 mM NaCl, pH 7.0 at 298 K. The reference titration of small molecules in the buffer was subtracted from the experimental data, and the data were fitted using the Origin 7.0 (OriginLab Corporation) software.

## Additional Information

**How to cite this article**: Liu, S. *et al.* Structure-based Mechanistic Insights into Terminal Amide Synthase in Nosiheptide-Represented Thiopeptides Biosynthesis. *Sci. Rep.* **5**, 12744; doi: 10.1038/srep12744 (2015).

## Supplementary Material

Supporting Information

## Figures and Tables

**Table 1:** Summary of diffraction data and structure refinement statistics.

	Se-NosA_1-111_
Summary of diffraction data	
Wavelength (Å)	0.9794
Space group	*P*4_1_32
Cell parameters	
*a* = *b* = *c* (Å)	143.3
Resolution (Å)	50.0-2.4 (2.59-2.40)^a^
Observed reflections	1,483,177
Unique reflections (I/σ(I) > 0)	18,105
Average redundancy	81.9 (81.8)
Average I/σ(I)	90.0 (17.6)
Completeness (%)	100.0 (100.0)
*R*_merge_ (%)^b^	9.2 (46.4)
Refinement and structure model	
Reflections (*Fo ≥ 0*σ*(Fo*))	
Working set	17,056
Test set	915
*R* factor/Free *R* factor (%)^c^	17.9/21.5
No. of protein atoms	2,207
No. of water atoms	128
Average B factor (Å^2^)	
All atoms	48.4
Main chain/side chain	46.6/51.4
Water	40.3
RMS deviations	
Bond lengths (Å)	0.007
Bond angles (°)	1.0
Ramachandran plot (%)	
Most favoured regions	96.6
Allowed regions	3.4
Generously allowed regions	0.0

^a^Numbers in parentheses represent the highest resolution shell.

^b^Rmerge = ∑_hkl_∑_i_|I_i_(hkl)_i_− < I(hkl) > |/∑_hkl_∑_i_I_i_(hkl).

^c^R = ∑_hkl_||F_o_|−|F_c_||/∑_hkl_|F_o_|.
