# Supplementary material for: Structure-based Mechanistic Insights into Terminal Amide Synthase in Nosiheptide-Represented Thiopeptides Biosynthesis
Source: Sci Rep. 2015 Aug 5;5:12744. doi: 10.1038/srep12744 (PMC4525488; doi:10.1038/srep12744)

Structure-based Mechanistic Insights into Terminal Amide Synthase in Nosiheptide-Represented Thiopeptides Biosynthesis

Shanshan Liu[1], Heng Guo[1,+], Tianlong Zhang[2,+], Li Han[1,+], Pengfei Yao[1,+], Yan Zhang[1], Naiyan Rong[1], Yi Yu[1], Wenxian Lan[1], Chunxi Wang[1], Jianping Ding[2], Renxiao Wang[1], Wen Liu[1]*, Chunyang Cao[1]*

Supporting materials

Fig. S1 The stability of full-length NosA and its truncated variants. (A) SDS-PAGE gel running for full-length NosA, lanes from 1 to 6, representing samples kept after 5 days, 10 days, 15 days, 20 days, 25 days and 30 days, respectively; (B) SDS-PAGE gel for $NosA_{1-140}$, lanes 1-2, representing samples taken before and after running the 1st Ni^{2+} column for purification, respectively; lane 3, representing the fractions collected on the 2nd column after digestion by thrombin protease; (C) SDS-PAGE gel for $NosA_{1-130}$, lanes 1-2, representing samples taken before and after running the 1st Ni column for purification; (D) SDS-PAGE gel for $NosA_{1-120}$, lanes 1-2, representing samples taken before and after running the 1st Ni^{2+} column for purification, respectively; lane 3, representing the fractions collected on the 2nd Ni^{2+} column after digestion by thrombin protease; (E) SDS-PAGE gel for $NosA_{1-111}$, lanes 1-4, representing samples kept after 5 days, 10 days, 15 days and 25 days, respectively. In all SDS_PAGE gels (A)-(E), lane M means protein marker, where the molecular weights were labeled. Same protein marker was used in running SDS_PAGE gels (B)-(E).

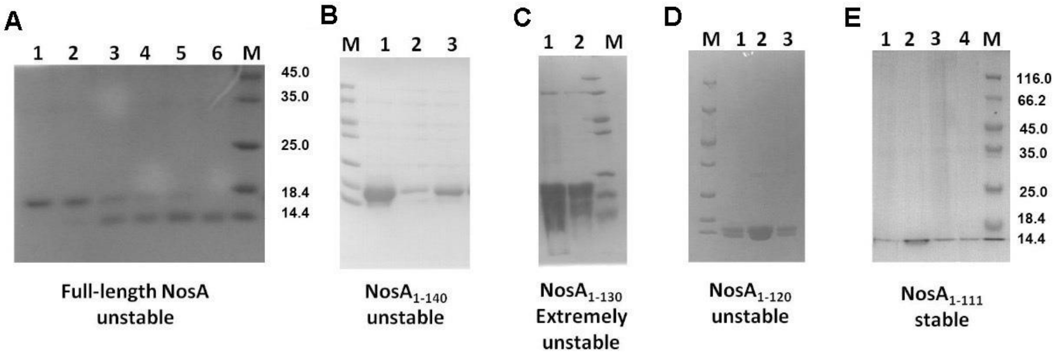

Fig. S2 The secondary structure of NosA predicted by Jpred 3 sever, where arrows indicate β-sheets, while barrels represents α-helices. The C-terminus of NosA (residues 106-151) was predicted as a flexible loop.

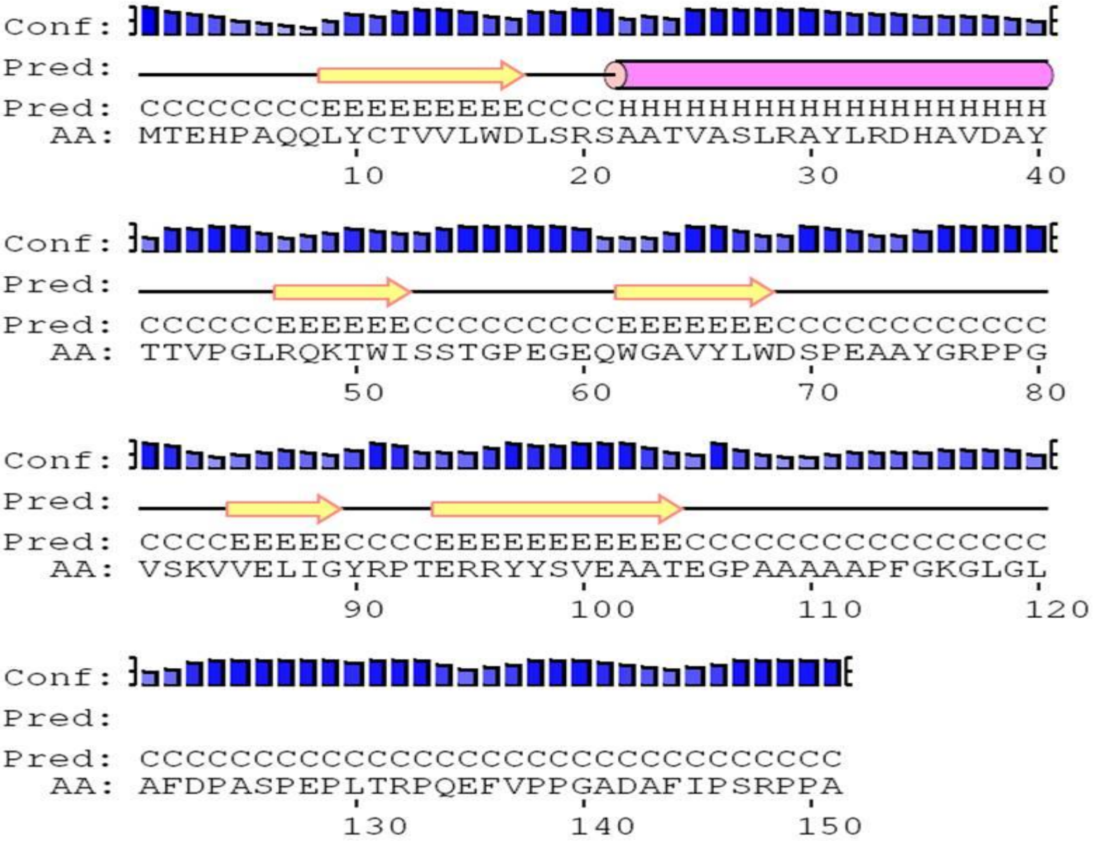

Fig. S3 The possible substrate binding sites of NosA predicted by comparison to its structural homologs. (A) Ribbon representation of $NosA_{1\text{-}111}$ with highlighted active sites K49 and E101'. Two monomers were displayed in yellow and blue, respectively. The side-chains of active sites K49 and E101 in one monomer (or K49' and E101' in another monomer) were represented in stick mode. (B) ActVA-Orf6 in complex with NOM, pdb code 1N5V; (C) IsdG in complex with two hemes, pdb code 2ZDO; (D) IsdI in complex with two hemes, pdb code 4FNH; and (E) MhuD in complex with four hemes. From (B) to (E), the ligand NOM or heme was highlighted in red or in pink.

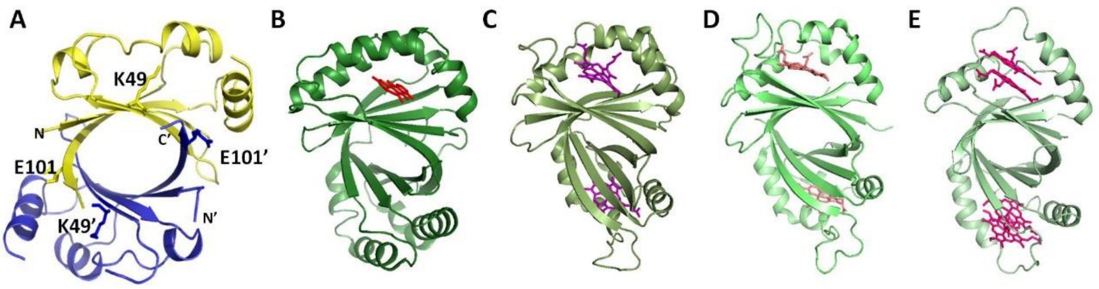

Fig.S4 Enzymatic assay on the full-length NosA and its variants running on HPLC system. In all cases, the substrate (the upper) and the product nosiheptide (the down) were used as controls, highlighted in a dotted red line and green line, respectively.

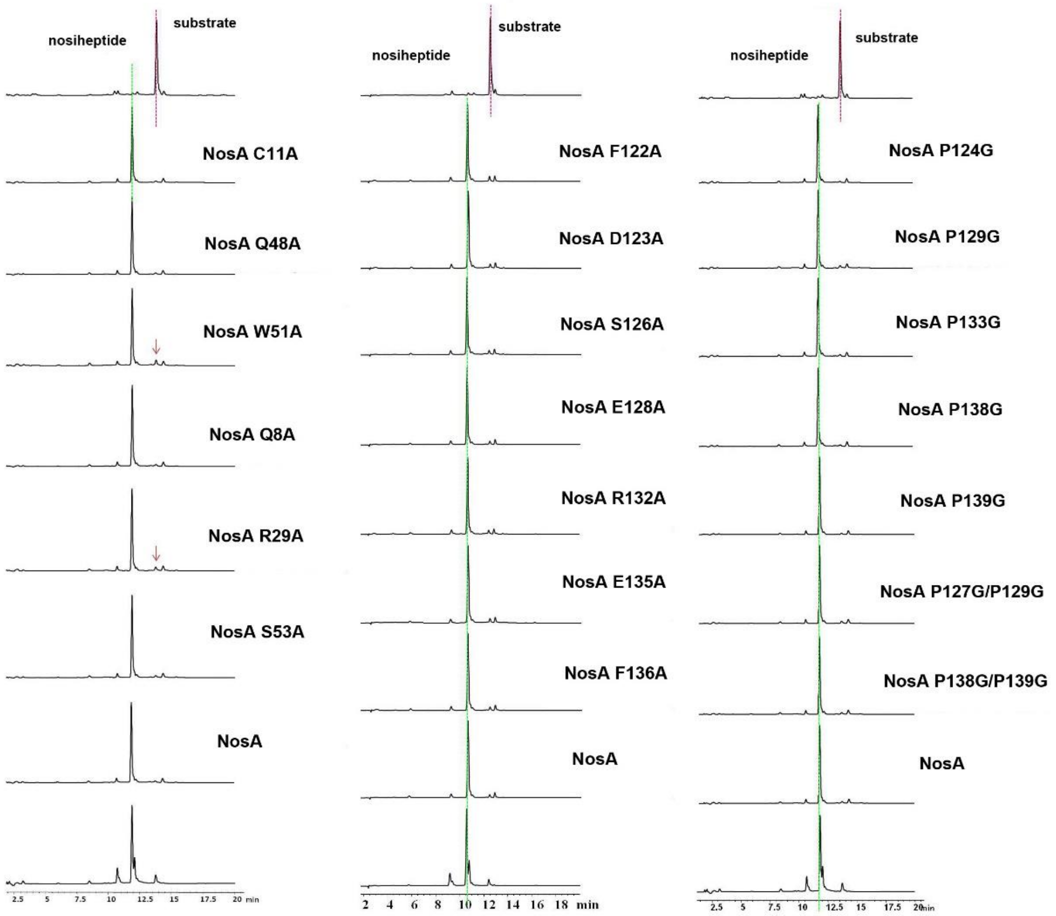

Figure S5 The distances measurement suggested that NosA may function as a dimer**.** The distances between the oxygen atoms of the –OH groups (highlighted in blue and red, respectively) and the C_{α}-N bond cleavage site in the substrate (left) were measured as 12Å and 20Å, respectively. The intra-molecular and inter-molecular distances between the oxygen atom in the side-chain of E101 and the nitrogen atom in the side-chains of K49 were measured as 26Å and 13.6Å, respectively. The intra-molecular and inter-molecular distances between the oxygen atom in the side-chain of E101 and the nitrogen atom in the side-chains of K49 were indicated by arrow-lines.

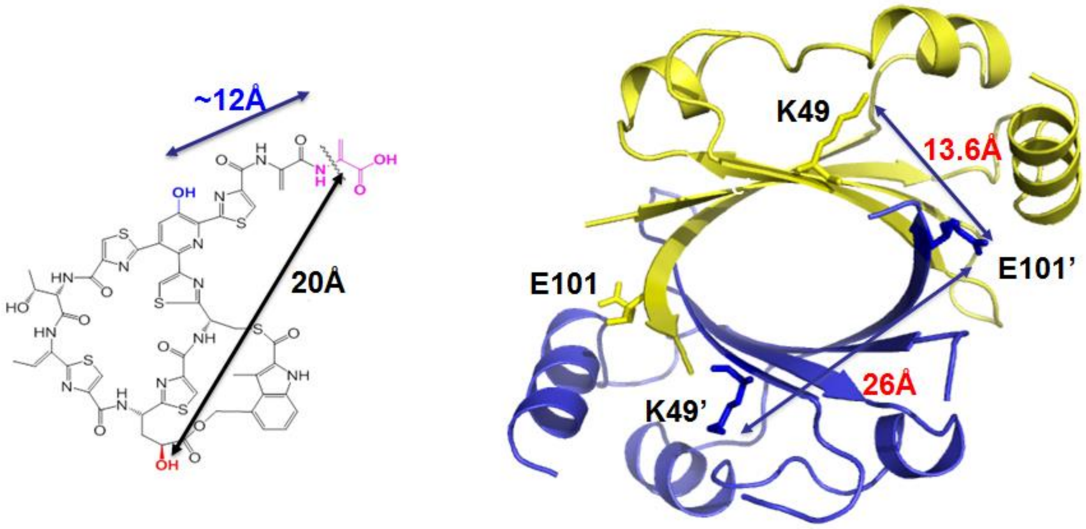

Fig. S6 The binding affinity of $NosA_{1\text{-}111}$ to $NosA_{112\text{-}151}$ measured by ITC assay.

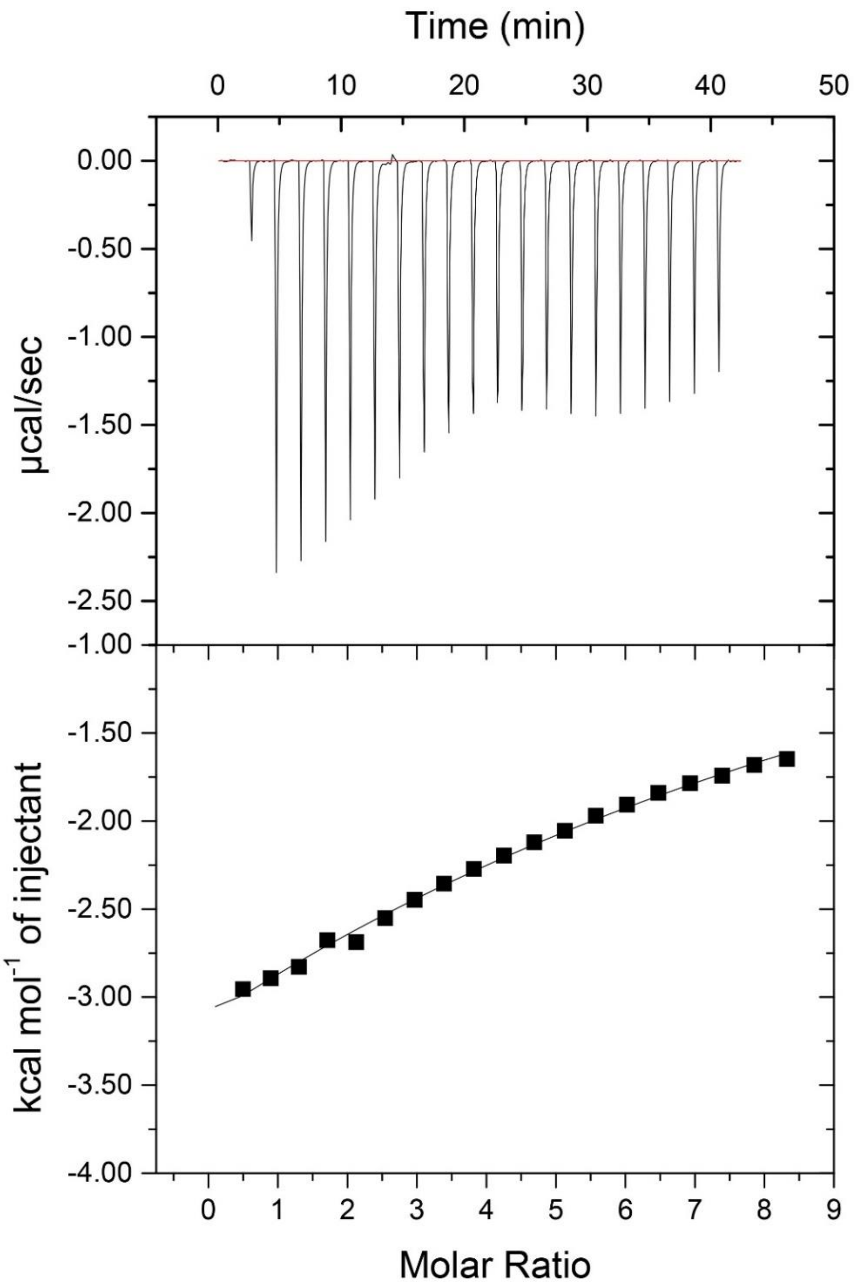

Fig. S7 $NosA_{112-151}$ is in random coil conformers in its free state and in the state of mixing with the N-terminal $NosA_{1-111}$ confirmed by (A) running circular dichroism (CD) spectrum of free $NosA_{112-151}$, and (B) by running ^{1}H-^{15}N HSQC spectrum on free $NosA_{112-151}$ (pink), overlapped very well with that acquired on the $NosA_{112-151}$ in complex with $NosA_{1-111}$ (cyan). The concentration of $NosA_{112-151}$ was about 0.2 mM in NMR buffer. And the mole ratio ($NosA_{1-111}$ vs $NosA_{112-151}$) =1:1.

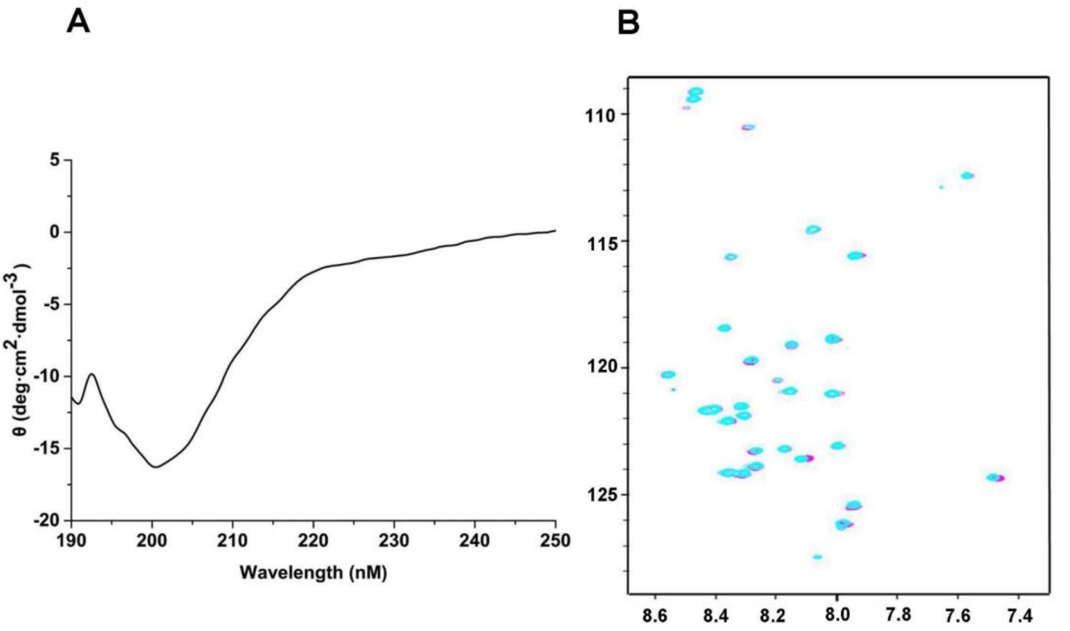

Fig. S8 Representative snapshots of the MD simulation trajectory. The N-terminal $NosA_{1-111}$ was demonstrated in a yellow ribbon mode, while the C-terminal $NosA_{121-140}$ fragment (121-140 aa) was displayed in blue ribbon mode. The substrate was highlighted in cyan-stick mode.

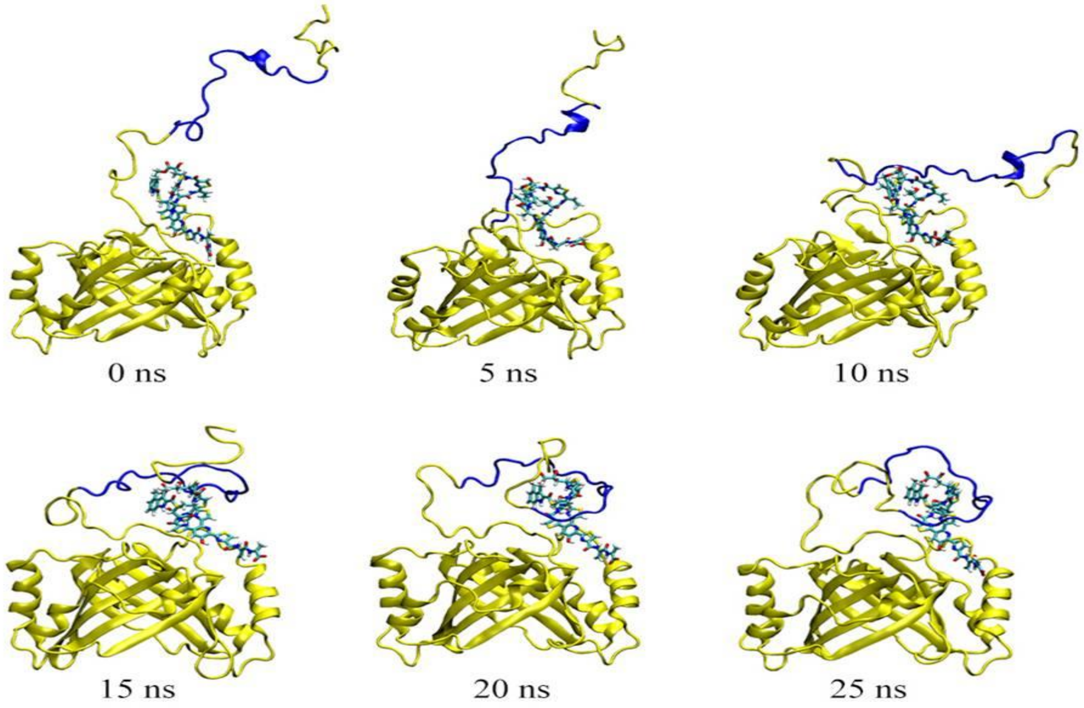

Supplement: Supporting Information [file srep12744-s1.pdf]
